# GE11 Peptide as an Active Targeting Agent in Antitumor Therapy: A Minireview

**DOI:** 10.3390/pharmaceutics10010002

**Published:** 2017-12-22

**Authors:** Ida Genta, Enrica Chiesa, Barbara Colzani, Tiziana Modena, Bice Conti, Rossella Dorati

**Affiliations:** Department of Drug Sciences, University of Pavia, Viale Taramelli 12, 27100 Pavia, Italy; enrica.chiesa01@gmail.com (E.C.); barbara.colzani@unipv.it (B.C.); tiziana.modena@unipv.it (T.M.); bice.conti@unipv.it (B.C.); rossella.dorati@unipv.it (R.D.)

**Keywords:** drug targeting, antitumor drug, GE11, EGFR, colloidal drug delivery systems, nanomedicine

## Abstract

A lot of solid tumors are characterized by uncontrolled signal transduction triggered by receptors related to cellular growth. The targeting of these cell receptors with antitumor drugs is essential to improve chemotherapy efficacy. This can be achieved by conjugation of an active targeting agent to the polymer portion of a colloidal drug delivery system loaded with an antitumor drug. The goal of this minireview is to report and discuss some recent results in epidermal growth factor receptor targeting by the GE11 peptide combined with colloidal drug delivery systems as smart carriers for antitumor drugs. The minireview chapters will focus on explaining and discussing: (i) Epidermal growth factor receptor (EGFR) structures and functions; (ii) GE11 structure and biologic activity; (iii) examples of GE11 conjugation and GE11-conjugated drug delivery systems. The rationale is to contribute in gathering information on the topic of active targeting to tumors. A case study is introduced, involving research on tumor cell targeting by the GE11 peptide combined with polymer nanoparticles.

## 1. Introduction

Drug targeting relevance is increasing as long as the knowledge about cellular targets and precise targeting agents increases. A rational targeting is able to significantly improve antitumor therapy, thus decreasing the adverse effects and improving the bioavailability of chemotherapeutics. Moreover, drug targeting can help to overcome the development of drug resistance which is one of the main causes of therapeutic failures [1,2]. Cancer chemotherapy is preferentially performed via parenteral administration. Better refined delivery of drugs via intravenous and interstitial administration routes remains at the forefront of research efforts, where nanomedicine plays an utmost relevant role [3,4,5,6,7].

Nanotechnology has the potential to create new materials and devices with a wide range of applications. In the pharmaceutical field, nanoparticles (NPs) made of biodegradable and biocompatible polymers present several advantages as carriers for therapeutics, such as the ability to encapsulate a wide variety of agents, including peptides, proteins, and genes, and to control drug release rates. The latter property is particularly important when administering chemotherapeutics, because a strict control of drug release and target release can be beneficial in reducing drug toxicity and improving drug efficacy. Therefore, polymer NPs result to be useful in treating severe and harming pathologies such as cancer and immunological diseases. NPs potential benefits in the diagnosis and treatment of metastatic cancer include their ability to transport complex molecular cargoes to the major sites of metastasis, such as the liver, lungs, and lymph nodes. Targeted polymeric NPs can be obtained by the synthesis of hybrid or biointegrated nanosystems where the combination of polymers with biomolecules, such as peptides, proteins, or monoclonal antibodies offers opportunities for the design of precise and versatile nanoscale systems. This can be achieved by adsorption, conjugation, or encapsulation of biomolecules in polymeric materials. The nanoscale system composition can properly tune cells uptake and further allows to control drug pharmacokinetics, as well as its activity and safety. The chemical conjugation of polymers to proteins and peptides seems to offer increased ability to precisely engineer NPs surface and represents a promising approach to reproducibly formulate targeted NPs. The central challenge of these smart materials is represented by the optimal interplay of biologic and physicochemical parameters in order to confer molecular targeting, immune evasion, and optimal drug release. Moreover, the ability to overcome physiological barriers in vivo is another important challenge of smart NPs. From the synthetic standpoint, the development of prefunctionalized biomaterials composed by all the desired NPs components and their engineering for self-assembly into targeted NPs, eliminate the need of particle postmodification. Prefunctionalized biomaterials result in high-precisely engineered NPs. Nevertheless, simpler conjugation and purification procedures are amenable to scale-up with little batch-to-batch variability.

Briefly, so far, a variety of innovative colloidal, multifunctional drug delivery systems (DDS) have been investigated for anticancer drug delivery. From the structural standpoint, the carriers can be: liposomes, polymeric microparticles (size > 1 μm), polymer nanoparticles (size < 800 nm), metal nanoparticles, solid lipid nanoparticles (SLN), polymer conjugated, dendrimers, lipoplexes [8,9,10,11,12,13,14,15,16,17,18,19]. From the functional standpoint, they are classified as first-, second-, and third-generation DDS. First-generation DDS include polymer microspheres for controlled drug release. They are: (i) depot formulations such as Zoladex and Leupron Depot, on the market for use in prostate and hormone-dependent cancers; (ii) colloidal formulations such as liposomes and stealth liposomes (PEGylated liposomes) for doxorubicin delivery. Doxil and Caelyx were the first liposomal formulations FDA-approved as anticancer DDS [1]. Paclitaxel-conjugated albumin nanoparticles, such as Abraxane, are approved for metastatic breast cancer [19].

Accordingly, first-generation DDS were designed to exploit the passive distribution due to the typical, enhanced permeation and retention effects (EPR) of tumor tissues. The high permeability of the capillaries in tumor tissues is due to proangiogenic factors that induce the proliferation of vessels with an incomplete endothelium. The phenomenon allows for the preferential accumulation of colloidal systems in these compartments. The passive distribution of nanocarriers is affected by: (i) physiological parameters, e.g., cardiac output; (ii) nanocarrier size and surface properties; (iii) release kinetics of the drug from the nanocarriers; (iv) endothelial characteristics and alterations [20]. Second-generation DDS are active tumor-targeting DDS obtained by exploiting molecules that recognize biostructures (receptors, antigens etc.) on the tumor cells.

Targeting agents can be: endogenous factors, lipoproteins, cytokines, hormones, growth factors, metabolites, oligonucleotides etc. [21,22,23,24]. Third-generation DDS combine the typical features of first-generation DDS (size and stability) and second-generation DDS (active targeting) with functional modules. They allow for displaying complex operations through consequent logical events such as: transport through biological barriers, disposition in the target tissue, selective cell recognition, cell uptake, masking–demasking events, and controlled drug release. Selective DDS disposition can be also obtained by the application of external stimuli such as magnetic fields [25,26].

Due to the overwhelming literature on nanotechnology in cancer therapy and targeting colloidal DDS, this minireview will focus on a specific topic, that is, the active targeting to tumors achieved by GE11 peptide combined with nanoparticulate DDS.

## 2. EGFR and EGFR Targeting

**Figure 1:**
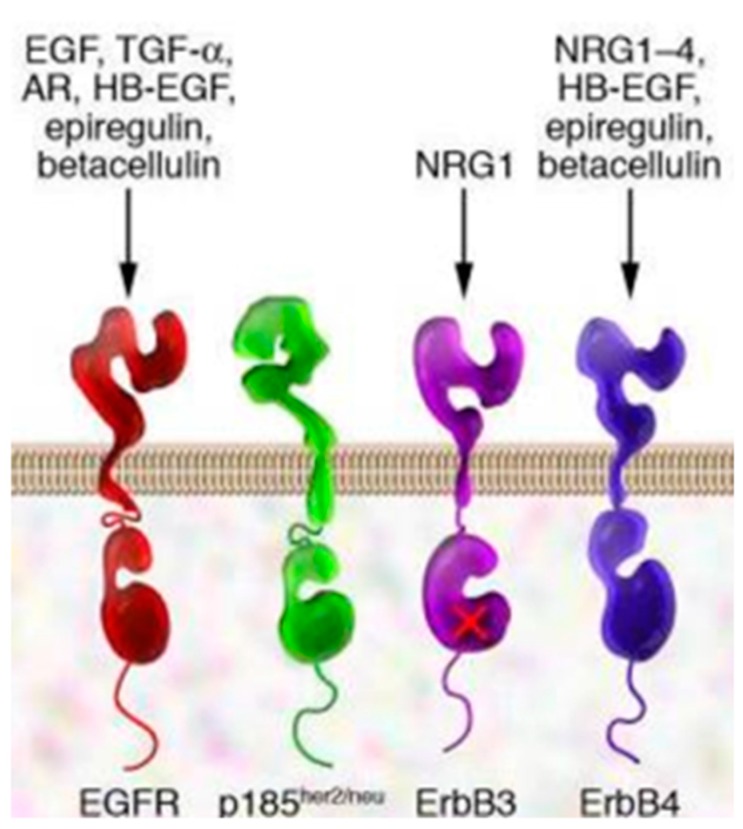
Epidermal growth factor receptors(EGFR), p185 (neu oncogene originally identified in cell lines derived from rat neuroectodermal tumors. neu is related to but distinct from the c-erbB gene, which encodes the epidermal growth factor (EGF) receptor. It encodes a protein, designated p185, that is serologically related to the EGF receptors), ErbB3 (receptor tyrosine-protein kinase, is a membrane bound protein that in humans is encoded by the ERBB3), ErbB4 (receptor tyrosine-protein kinase, is an enzyme that in humans is encoded by the ERBB4 gene). EGFR specific ligands: transforming growth factor alfa (TGF-α), androgen receptor (AR), Neuregulin 1 (NRG1, NRG1-4), heparin-binding EGF-like growth factor (HB-EGF).

A lot of solid tumors are characterized by uncontrolled signal transduction triggered by receptors related to cellular growth. The epidermal growth factor receptor (EGFR) family (ErbB1, EGFR, etc. Figure 1) is a family of receptors highly involved in this mechanism [27,28,29,30,31].

The receptor role was discovered in 1984 by Drebin, who observed the presence of a tumor antigen on the surface of cells expressing the oncogene neu, which is a 185 KDa protein belonging to EGFR family. The expression of this receptor type was highlighted in breast primary tumors with negative prognosis [28]. Further mutations, involving another receptor of the same family, are evident in non-small cell lung cancer (NSCLC). Genetic alterations of this receptor family are responsible for receptor overexpression, change of the receptor intracellular enzymatic portion, simultaneous expression of the receptor and its ligands [32]. All these cases contribute to activate the intracellular pathway related to the receptor. Herbst and co-workers [33] evaluated the percentage of increased EGFR expression within some tumor types. The authors showed that tumors such as head and neck, renal, and lung cancer, are highly affected by EGFR overexpressing mechanisms, in percentages between 80–100% for head and neck cancer, 50–90% for renal cancer, and 40–80% for lung cancer.

EGFR shows tyrosine kinase receptor activity, which is essential for cell growth, proliferation, survival, and differentiation [27,34]. Diverse types of EGFR belong to this family, namely, EGFR/Herb1, HER2/Erb2, HER3/Erb3, HER4/Erb4 (Figure 1). Entering some details, EGFR are transmembrane receptors characterized by an extracellular portion which recognizes specific ligands (i.e., EGF, EGF-like molecules) and an intracellular portion with tyrosine kinase enzymatic activity. The isolated receptor monomers are dormant. When specific ligands bind to the receptor extracellular portion, it dimerizes generating homodimers and heterodimers.

The dimerized receptor triggers the receptor enzymatic activity through the phosphorylation of tyrosine residues in the receptor C-terminal portion, resulting in intracellular signal transmission activation. Mitogen-activated protein kinase (MAPK) and Phosphoinositide 3-kinase/protein kinase B are the most important effectors. The final effect of these intracellular pathways is always an increased transcription of genes responsible for cell growth [28]. The receptor tumoral forms, such as Erb2, are in their extended conformation after ligand-mediated activation, and they can dimerize even in the absence of exogenous ligands. Signal transduction stops after the endocytosis of the ligand–receptor complex, followed by either its degradation, or its new expression at the cell membrane.

Considering the importance of these receptors in the development of several tumor types, these molecules are the target of several therapeutic strategies studied as an alternative or as a completion and improvement of traditional chemotherapy, especially when resistance to traditional chemotherapy is highlighted.

For example, anti-EGFR IgG monoclonal antibodies are successfully experimented molecules, with medicinal products on the market such as Herceptin^®^ (Trastuzumab) and Erbitux^®^ (Cetuximab). However, monoclonal antibodies scarcely penetrate into solid tumors and have high manufacturing costs. For these reasons, monoclonal antibodies fragments were introduced despite their lower receptor affinity and half-life [35].

The inhibition of the tyrosine kinase activity of the EGFR receptor C-terminal portion represents another therapeutic approach for some EGFR-dependent tumors. In this case, small molecules are used. They can be either ATP antagonists in tyrosine residue phosphorylation, or molecules promoting chemical irreversible alkylation. These molecules have the advantage to recognize all EGFRs independently of their subtype. Some examples of products on the market containing reversible inhibitors of tyrosine kinase activity are Tarceva^®^ (Erlotinib), used in non-small cell lung cancer therapy, and Iressa^®^ (Gefitinib). A disadvantage of the reversible inhibitors is represented by resistance development due to genetic mutations, with loss of drug activity. Irreversible inhibitors, such as Giotrif^®^ (Afatinib), used in NSCLC therapy, do not have this drawback.

Summarizing, the EGFR family includes cellular transmembrane receptors with tyrosine kinase enzymatic activity. Its positive signalling causes increased cell proliferation, decreased apoptosis, enhanced cell motility, and angiogenesis. EGFR overexpression is typical of tumors of epithelial origin, such as breast, colorectal, and lung cancer [36,37]. Recent studies demonstrate that EGFR is involved also in other severe pathologies, such as rheumatoid arthritis, which is characterized by aberrant cell growth. Swanson and co-workers [37] demonstrated EGFR overexpression in synovial fibroblasts and endothelial cells, both in patients suffering from rheumatoid arthritis and in a murine model of the pathology. Therapies available on the market already approach the EGFR targeting strategy [38], showing this is a winning strategy that can be improved by new targeting molecules and their combination with DDS. A goal is to exploit DDS decorated with active targeting agents (smart DDS) in order to improve the therapeutic efficacy of old chemotherapeutics.

## 3. GE11 Structure and Biologic Activity

Peptides represent a suitable alternative to monoclonal antibodies as active targeting agents [39,40]. They are studied for DDS functionalization with the goal to achieve smart DDS. They have low immunogenic potential and show good penetration into solid tumor tissues. Moreover, peptides can be easily synthesized and conjugated to polymers [41]. Despite the lower affinity of peptides with respect to monoclonal antibodies, the high density of peptide decoration that can be achieved on nanoparticles surface warranties high targeting results. Peptides active targeting agents are preferred to EGF endogenous ligands because the latter stimulates EGFR activation, triggering cell growth and proliferation. For this reason, the research for active targeting agents to be combined with DDS has focused on small molecular weight peptides with good affinity for EGFR.

**Figure 2:**
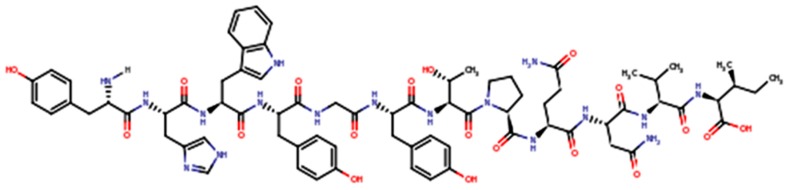
GE11 primary structure.

GE11 (YHWYGYTPQNVI, Mw (Molecular weight) 1540 g/mol, IP 7.67, Figure 2) is a dodecapeptide whose excellent EGFR affinity was demonstrated [40].

GE11, with its affinity towards EGFR, was identified by Li and colleagues [39] through the screening of phage display libraries (phage display technique). GE11 affinity for EGFR was demonstrated to be significantly higher (kd = 22 nM) than that of Bovine Serum Albumin (BSA). GE11 is a small peptide with 12 amino acids. It is much smaller than EGF, and it binds only to one EGFR region. Therefore, GE11 has a relatively lower affinity for EGFR (kd = 22 nM) than EGF (kd = 2 nM). GE11 moves from EGFR after the addition of the physiologic ligand EGF, demonstrating both its selective binding to EGFR and its receptor affinity. As said, the mentioned studies demonstrated that GE11 has a lower affinity than the EGF physiologic ligand, but the peptide does not have mitogen activity. Recent literature reports that GE11 has a high potential to accelerate nanoparticle endocytosis, and that this is due to an alternative EGFR-dependent actin-driven pathway. The authors showed that the EGFR level on the surface of cancer cells remains constant after treatment with GE11 polyplexes, indicating an EGFR recycling process with a prolonged receptivity of the cells for circulating GE11 polyplexes [41,42].

**Figure 3:**
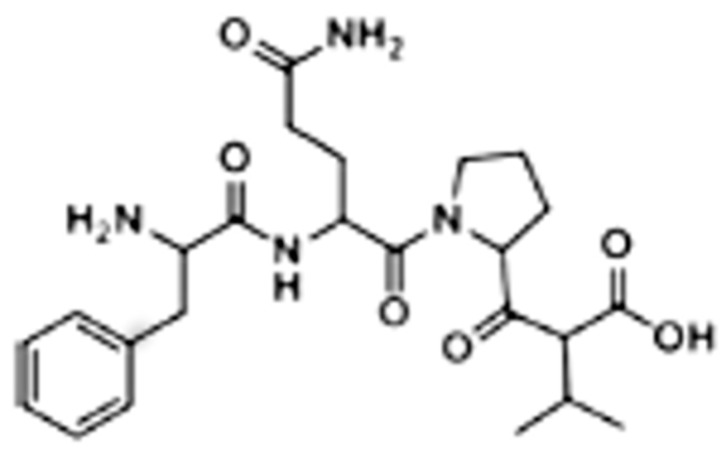
Primary structure of the FQPV tetrapeptide.

The GE11 peptide can be easily synthesized by microwave-assisted solid phase automated synthesis. Colzani and co-workers used a BiotageSP Wave Initiator synthesizer, Fmoc-protected amino acids, and a preloaded Fmoc Ile-2-Cl-Trityl resin swelled with dimethylformamide [43]. They obtained peptides whose purity was higher than 95%, as determined by analytical HPLC. The authors’ strategy was to synthesize a small tetrapeptide before performing GE11 synthesis, in order to optimize the reaction protocol. The FQPV tetrapeptide, whose structure is reported in Figure 3, was selected as a model. The synthesis was carried out on a 0.2 mmol scale (450 mg loaded resin).

## 4. GE11 Conjugation and GE11-Conjugated Drug Delivery Systems

Results in the literature confirm GE11 to be an excellent allosteric EGFR ligand and address studies focused on GE11 use as an active targeting agent for EGFR-overexpressing solid tumors. GE11-targeted drug delivery systems include liposomes, polymer-based polyplexes, and filamentous plant viruses based or polymeric nanoparticles for diagnostic as well as anticancer and gene delivery applications. Researches are mostly addressed to obtain long-circulating nanocarriers which exploit a targeting potential derived from the presence of the GE11 peptide on their surface. In this way, GE11 is exposed to EGFR targeting. Stable and long-circulating carriers are needed to ensure that the drug and the targeting agent are not released before the nanocarrier has reached its target. Pharmacodynamic and pharmacokinetic studies are important on this purpose. Nanocarriers long-circulating properties can be obtained and modulated by including polyethylenglycol (PEG) in their composition. This is a well-known strategy to achieve stealth nanocarriers. The presence of PEG on the nanocarrier surface prevents its interaction with plasma opsonin and avoids or reduces the nanocarrier uptake by macrophages. GE11 can be reacted with pegylated polymers or phospholipids in order to achieve stable conjugates. Several studies on GE11–PEG conjugates were published, where the peptide was directly bound to the PEG block of copolymers, such as GE11 conjugated to pegylated poly-lactide-co-glycolide (GE11-PEG-PLGA), pegylated poly-ε-caprolactone (GE11-PEG-PCL), gelatin (GE11-PEG-gelatin), or 1,2-distearoyl-sn-glycero-3-phosphoetahnolamine (DSPE-PEG-GE11) [30,44,45,46,47,48,49,50,51,52,53,54].

GE11 surface-decorated nanoparticles can be obtained mainly by the following pathways: (i) conjugation of GE11 to the polymer or copolymer and subsequent preparation of nanoparticles using the conjugated polymer (or copolymer); (ii) preparation of polymer nanoparticles and subsequent conjugation by surface interaction of the polymer with GE11.

As reported, most used polymers are biodegradable and biocompatible, such as poly-alfa-hydroxyacids, polylactide (PLA), polylactide-co-glycolide (PLGA), polyε-caprolactone (PCL), and the pegylated copolymers of these polymers. They are well known in the scientific community for being biodegradable and biocompatible and they are approved by FDA and EMA regulatory agencies for use in injectable products [55,56]. Injectable medicinal products, namely, DDS based on these polymers, are already on the market for cancer therapy (e.g., Enantone depot, Lupron etc.). All these advantages make these polymers very popular in studies focused on smart DDS.

PLA, PLGA, PCL, and pegylated PLA, PLGA, and PCL are either synthesized on purpose with suitable Mw, or already available on the market. GE11–PEG conjugation can be performed starting from the pegylated copolymers. Moreover, the pegylated copolymers are amphiphilic block copolymers with several advantages: (i) when processed by nanoprecipitation or the emulsification method in an aqueous environment, they spontaneously form nanoparticles with core–shell structure; (ii) PEG hydrophilic blocks preferentially arrange on the nanoparticles surface, forming PEG surface-decorated nanoparticles; (iii) PEG corona creates a hydrophilic steric barrier that increases in vivo the nanoparticles circulating time because it prevents nanoparticle aggregation in plasma and delays opsonization and recognition by the Reticulo Endotelial System (RES) [57,58].

**Figure 4:**
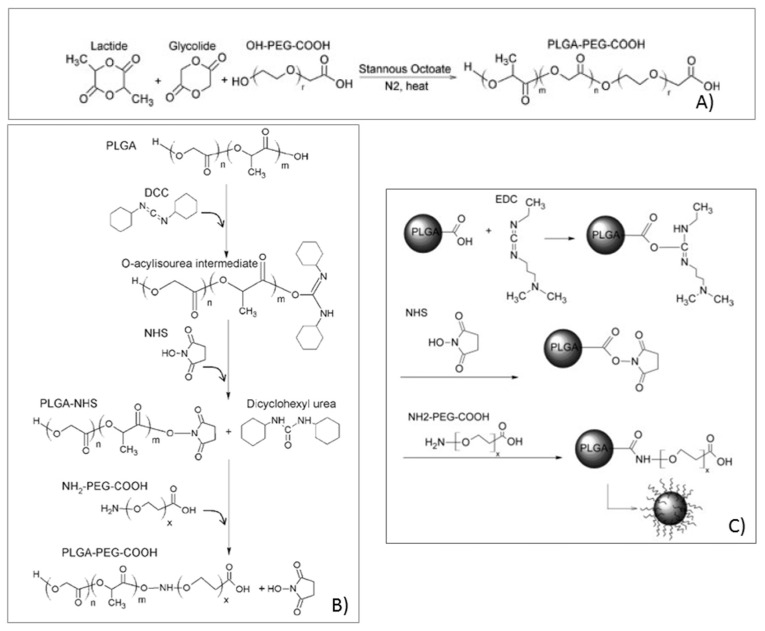
Polyethylenglycol (PEG) conjugation to the polylactide-co-glycolide (PLGA) polymer: (**A**) PEG conjugation throughout the PLGA polymerization reaction; (**B**) PEG conjugation to premade PLGA; (**C**) PEG conjugation to previously formed nanoparticles. (Modified from Bentacourt and co-workers. 2009 [57]).

Pegylation of poly-alfa-hydroxyacids can be performed by various methods. As an example, Bentacourt and co-workers [59,60] performed PLA and PLGA pegylation by means of eterofunctional OH-PEG_3400_-COOH containing hydroxyl and carboxyl end groups. The authors explored three PEG conjugation methods: (i) PEG conjugation during PLGA or PLA polymerization synthesis; (ii) PEG conjugation on preformed PLGA or PLA polymers; (iii) PEG conjugation on preformed PLGA or PLA nanoparticles. The first method involved the synthesis of PLGA or the PLA–PEG block by ring opening polymerization of cyclic glycolide and, or, lactide dimers initiated by the hydroxyl end groups of PEG and in the presence of Sn octoate as a catalyzer, as schematized in Figure 4A. The polymerization reaction takes place in anhydrous conditions with toluene at 110 °C, under pressure (20 psi) in a sealed environment, and in the presence of N_2_. The amount of PEG and dimers are calculated from the molecular weight and composition of the desired final copolymer, taking into account that each PEG hydroxyl end group initiates the polymerization of a single PLGA or PLA chain. When polymerization is terminated, the reaction flask is cooled-down in iced water, and the polymer is precipitated with ethyl ether and centrifuged. The obtained pellets are dissolved in methylenchloride, vacuum-dried in a dessicator and freeze-dried (−20 °C). This method achieved good results in terms of yield of production of the copolymers. The control of the reaction conditions permitted to obtain copolymers with selected composition and Mw. The copolymers obtained were stable and it was demonstrated by H^1^NMR and Gel Permeation Chromatography (GPC) that the copolymer composition and Mw were maintained even after nanoparticle preparation. In another example from Bentacourt and co-workers [59], premade PLGA terminated in carboxyl and hydroxyl groups was conjugated to heterofunctional NH_2_-PEG-COOH utilizing standard carbodiimide/NHS-mediated chemistry, as shown in Figure 4B. PLGA was reacted with *N*,*N*-dicyclohexyl carbodiimide (DCC). *N*-hydroxysuccinimide (NHS) at 23 °C was used to activate the carboxylic acids to the semistable amine-reactive activated NHS ester of PLGA. After a stated time, the polymer mixture was filtered to remove the insoluble dicyclohexil urea byproduct. The solution was concentrated, and the polymer was eventually precipitated with ethyl ether or methanol (−20 °C). The polymer was pelleted by centrifugation and the supernatant discarded. A further copolymer purification was performed to separate the dicyclohexyl urea byproducts. PLGA–NHS was reacted with NH_2_-PEG-COOH in an organic solvent and in the presence of *N*,*N*-diisopropylethylamine to desalt the terminal PEG amine group. After the reaction, the solution was concentrated and the polymer recovered and purified by three cycles of precipitation and centrifugation. The purified copolymer pellet was dried under vacuum, frozen, and freeze dried. This conjugation method resulted in a moderate conjugation efficiency depending on the specific conditions in which the conjugation was carried out. The main difficulty encountered with this method derived from the cumbersome purification steps by precipitation of the copolymer that prevented to recover high yields. The different process conditions and parameters used can affect PEG conjugation efficiency.

Similarly, carbodiimide chemistry was used to conjugate PEG to already formed PLGA nanoparticles, following the scheme reported in Figure 4C.

The three studied methods gave significantly different results, and only pegylation, carried out throughout a polymerization reaction (first method), resulted to be satisfactory in yield and pegylation efficiency.

Some examples of studies on GE11–DDS formulations are reported and discussed here below.

Master and co-workers [61] prepared and characterized polymer micelles made of Polyethylenglycol-polyε-carpolactone as carriers for PC4, a photosensitive agent used for photodynamic tumor treatment. The micelles surface was modified with GE11 to facilitate EGFR active targeting-mediated internalization. The micelles surface modification was obtained in two steps, as briefly explained here below.

Maleimide-functionalized polyethylenglycol-block-caprolactone was synthesized by reacting Mal–PEG-OH and ε-caprolactone in dry toluene at 130 °C for 3 days with Sn(Oct)_2_ as catalyst. The resulting functionalized polymer product was isolated by dissolution in tetrahydrofuran and subsequent precipitation in n-hexane. Cys-GE11 peptide and Mal–PEG–PCL were reacted in a 2:1 molar ratio in an aqueous buffer at room temperature for 4 h to allow the reaction of the Mal group with the sulphydril groups of cysteine residues to form a thioether linkage. The unreacted reagents and peptide were removed by dialysis against Millipore water. The conjugation was confirmed by H^1^NMR. In vitro uptake studies were perfomed on tumor cell overexpressing EGFR and showed that GE11-targeted polymer micelles were uptaken significantly faster with respect to the micelles without GE11 linkage. The presence of GE11 on the micelles surface activated an active uptake mechanism involving ligand-mediated endocytosis, while the cell uptake of nontargeted micelles happend only by passive diffusion. In vivo studies demonstrated the faster cellular uptake of GE11 micelles with a prolonged effect [62].

Milane and co-workers modified GE11 C-termini with a GGGGC sequence in order to achieve PEG–PLGA functionalization with GE11. Indeed, they demonstrated that the peptide far ends are not needed for GE11 activity. The peptide–polymer conjugate was mixed with PCL and manufactured in polymer micelles with a hydrophobic core and a hydrophilic shell made of PEG and GE11. The nanoparticles loaded with Ionidamine and paclitaxel demonstrated high selectivity for EGFR-overexpressing cell lines, with fast initial cellular uptake. The same formulations administered to a murine model of EGFR-overexpressing tumor did not show a significant difference between the GE11-targeted and nontargeted micelles as long as the uptake rate and effect persistence were concerned. The authors hypothesized that the in vivo results were due to the scarce presence of GE11 on the nanoparticle surface and to the variability of EGFR expression based on cell genotype. However, even if the in vivo results were inconsistent, active targeted nanoparticles contributed to improve the drug therapeutic effect also by reducing nanoparticle uptake into nontarget tissues [53,63,64].

Colzani and co-workers prepared nanoparticles made of a PLGA and a PEG–PLGA blend where GE11 was directly conjugated to PLGA. The authors aim was to obtain a nanoparticle platform where the nanoparticle hydrophilicity/hydrophobicity ratio was modulated by the polymer blend composition. The platform can be a useful tool in order to optimize the loading of chemotherapeutic drugs or the dual co-loading of drugs with different physicochemical properties. GE11 conjugation to PLGA was performed through PLGA activation with 1-ethyl-3-(3-dimethylaminopropyl)carbodiimide (EDC) and NHS, following soaking of the activated PLGA with GE11 in acetonitrile at room temperature. The GE11–PLGA conjugate was desiccated with sodium sulfate, recovered by vacuum drying, and characterized by H^1^NMR. PLGA functionalization with GE11 resulted in a good process yield (>80%) and high degree of polymer functionalization with the bioactive moiety (>85%). The functionalization method is quite simple. The coupling reaction involves the native functional groups of both PLGA (carboxylic end group) and peptide (*N*-terminal amino groups), thus the probability to lose the ligand specific activity is low [44]. Starting from the GE11–PLGA conjugates, the authors prepared nanoparticles by a nanoprecipitation method and by blending PEG–PLGA to the GE11–PLGA conjugates. The procedure allows to select starting polymers with different molecular weights, therefore it ensures the ability to tailor the resulting nanoparticles behavior in terms of drug release rate and polymer degradation time. Moreover, the GE11–PLGA conjugate is more hydrophilic than the starting PLGA, and nanoparticles can be prepared using more ecofriendly and less toxic solvents, if compared to the commonly used halogenated solvents such as methylen chloride. The authors thoroughly characterized the nanoparticles for their stability, even in human plasma, using the fluorescence Single Particle Tracking (fSPT) technique, and demonstrated nanoparticles stability with lack of aggregation in human plasma. In vitro tests on cellular uptake were performed on fluorescently labeled GE11-PLGA/PEG–PLGA nanoparticles in incubation with A549 cells, a human lung carcinoma cell line overexpressing EGFR. They demonstrated nanoparticle fast uptake with stable intracellular levels for 24 h at 37 °C [64]. The study was focused on the formulation study of GE11-conjugated nanoparticles, especially investigating those aspects that could represent an issue in the development of a medicinal product.

Liposomes have been widely studied as drug delivery systems in cancer therapy, with successful examples already on the market, such as Doxil^®^. It is recognized by the scientific community that liposomes can be suitable carriers for anticancer drugs since they permit to reduce drugs toxicity and adverse effects. The phospholipid bilayer interacts well with cell membranes through the endocytic pathway, meanwhile protecting healthy tissues from a direct contact with the drug. Liposomes behavior can be tuned depending on phospholipid composition and their combination to PEG. Pegylated liposomes circulate longer time when injected in the body, therefore prolonging the drug effects. Liposomes carrying active targeting agents can overcome the challenge of poor drug transfer from liposome to tumor and significantly improve DDS efficacy. GE11 has been studied among the several targeting ligands introduced in the literature, for example for estrone and tamoxifen targeting in breast cancer, or folate [42,65,66,67,68]. Pegylated liposomes carrying GE11 were prepared either as drug carriers or as contrast agents [42,68]. The authors demonstrated that GE11 targeting efficiency and cellular uptake varied depending on the cell type and bioconjugate nature.

Xu and co-workers prepared dual therapeutic loaded GE11-conjugated liposomes following the therapeutic strategy of combining docetaxel with siRNA and GE11. siRNA was employed to decrease the multidrug resistance of the targeted laryngeal tumor, while GE11 actively targeted the liposomes to the tumor cells overexpressing EGFR. The classic anticancer drug Docetaxel was loaded into liposomes made of a combination of egg phosphatidylcholine (EPC), DSPE-PEG2000 (1,2-distearoyl-sn-glycero-3-phosphoethanolamine-*N*-polyethylenglycol2000), DSPE-PEG2000-MAL (1,2-distearoyl-sn-glycero-3-phosphoethanolamine-*N*-polyethylenglycol2000-maleimide), DOTAP (*N*-[1-(2,3-Dioleoyloxy)propyl]-*N*,*N*,*N*-trimethylammonium methyl-sulfate), and cholesterol. GE11 was conjugated to PEG in the already formed liposomes and ABCG2-siRNA was adhered on the liposomes surface by electrostatic interaction. The results on a murine animal model showed a significant improvement of drug efficacy with enhanced antitumor effect and specificity [69].

Also, GE11 conjugated to the antitumor drug doxorubicin (DOX) was investigated and resulted to have the potential to be a therapeutic agent for treating EGFR-overexpressing tumors. Fan and co-workers [70] reacted GE11 with doxorubicin (DOX) via a disulfide. The bond can be cleaved by reduced glutathione (GSH) in order to release the antitumor drug. The authors investigated the intracellular delivery and in vitro cytotoxicity of the GE11–DOX conjugate in comparison to DOX. The investigation was performed in high- (SMMC-7721) and low- (MCF-7) EGFR-expressing cancer cell models. They found that GE11–DOX accumulated at higher levels in SMMC-7721 cells than in MCF-7 cells, and that an EGFR pathway was involved in the transport of the conjugate. On the other hand, the cellular uptake of free DOX was not dependent on EGFR expression.

An interesting study comparing the EGFR targeting capacity of GE11 small silica nanoparticles (50 nm size) was recently performed by Zarscheler and co-workers [70]. The authors prepared EGFR-targeted fluorescent silica nanoparticles by conjugation of specific peptides (GE11 and D4 functionalized nanoparticles) or small camelid domain antibodies (sdAB-functionalized nanoparticles). The investigation was performed by way of the receptor RNA silencing technique, in milieus of different complexity by addition of increasing concentrations of human serum proteins. The authors highlighted the importance of evaluating in vitro nanoparticles behavior in realistic biologic milieus, because the dynamic interactions of functionalized nanoparticles with components of complex biologic fluids can dramatically modify their targeting efficiency in vivo. They observed an inhibition of the overall nanoparticles uptake with increased protein concentration. Moreover, in these experimental conditions, sdAB-functionalized nanoparticles resulted to be more efficient than GE11-functionalized nanoparticles. However, it should be taken into account that silica nanoparticles could behave differently with respect to polymer nanoparticles or liposomes.

Chen and co-workers combined hyaluronic acid, a natural targeting agent for CD44-overexpressing receptor cells, with GE11. They prepared multifunctional redox-sensitive nanogels by the inverse nanoprecipitation method and the catalyst-free tetrazole alkene photoclick reaction. The authors demonstrated enhanced cellular uptake of the nanogels in SKOV-3 cancer cells overexpressing both CD44 and EGFR [71].

Indeed, Haeri and co-workers designed thermosensitive liposomes functionalized with GE11 and Fab fragments of cetuximab in order to specifically and more efficiently bind EGFR-overexpressing cancer cells. They obtained positive targeting results with increased intracellular cargo release promoted by hyperthermia [72].

## 5. Conclusions

GE11 is studied as a peptide ligand, selectively recognizing EGFR for diagnostic and therapeutic purposes with respect to EGFR-overexpressing cancer cells. Different types of DDS and GE11 conjugates are proposed in the literature. The peptide was conjugated to PEG, PLGA, PLA, PCL, and the copolymer conjugates were manufactured in polymer nanoparticles or liposomes.

The colloidal systems decorated with GE11 on their surface are proposed either as carriers for old antitumor drugs, such as doxorubicin and camptotecine, or as diagnostics.

Demonstrated advantages are the small peptide Mw, conjugate stability, and good binding capacity to EGFR. Moreover, GE11 can be directly conjugated to doxorubicin, improving the drug therapeutic effect.

Few constraints are reported in the literature. For example, GE11 binding density on the polymer nanoparticle surface is strictly dependent on the GE11 conjugation methods, and it is scarce when GE11 conjugation is carried out on already formed nanoparticles.

Controversial results are reported depending on the nanoparticles composition. GE11-functionalized polymer NPs result to be stable in environments simulating in vivo conditions, while GE11-functionalized silica NPs dramatically modify their behavior in the presence of complex fluids simulating in vivo conditions. Therefore, NPs composition significantly affects their behavior because of the interactions with plasma components, such as proteins.

Recent works show that GE11 can be used as a synergist of other targeting agents in tumors overexpressing more than one membrane receptor, or in order to improve EGFR targeting. Therefore, GE11 seems to be a useful and versatile EGFR targeting agent to be further developed in pharmaceutical products.
